# Antibody responses to porcine reproductive and respiratory syndrome virus, influenza A virus, and *Mycoplasma hyopneumoniae* from weaning to the end of the finisher stage in fourteen groups of pigs in Ontario, Canada

**DOI:** 10.1186/s12917-021-02756-6

**Published:** 2021-02-17

**Authors:** Elana Raaphorst, Abdolvahab Farzan, Robert M. Friendship, Brandon N. Lillie

**Affiliations:** 1 Department of Pathobiology, University of Guelph, 50 Stone Road East, Guelph, ON N1G 2W1 Canada; 2 Department of Population Medicine, University of Guelph, 50 Stone Rd E, Guelph, ON N1G 2W1 Canada

**Keywords:** Porcine reproductive and respiratory syndrome, Influenza A virus, *Mycoplasma hyopneumoniae*, ELISA, Antibody response, Nursery diet, swine

## Abstract

**Background:**

Respiratory diseases are among the most important factors affecting swine farm productivity in Canada. The objectives of this study were to investigate antibody responses to porcine reproductive and respiratory syndrome virus (PRRSV), influenza A virus (IAV), and *Mycoplasma hyopneumoniae* (*M. hyopneumoniae*) from weaning to the end of the finisher stage on a subset of commercial swine farms in Ontario, Canada, and to examine the association between nursery diet and antibody responses.

**Results:**

Overall, older pigs were more likely to test seropositive for PRRSV and less likely to test seropositive for *M. hyopneumoniae* (*p* <  0.001). Pigs were more likely to test seropositive for IAV at weaning and the end of the grower and finisher stages compared to the end of nursery (*p* <  0.001). Pigs that were seropositive for IAV were more likely to test seropositive for both PRRSV and *M. hyopneumoniae* (*p* <  0.001). Two, 9, and 4 groups that had more than 20% of pigs seropositive to PRRSV, IAV, and *M. hyopneumoniae*, respectively, from the end of nursery to the end of finisher were classified as seropositive. Pigs fed a plant-based (low complexity) diet during nursery were more likely to be seropositive for PRRSV (*p* <  0.001) but there were no significant differences in seropositivity to IAV or *M. hyopneumoniae* due to nursery diet complexity.

**Conclusions:**

This study provides information regarding changes in serum antibody in pigs across different stages of production and highlights periods of vulnerability. Additionally, these findings may encourage further research into the effects of nursery diet complexity on disease susceptibility and immune response.

**Supplementary Information:**

The online version contains supplementary material available at 10.1186/s12917-021-02756-6.

## Background

Respiratory diseases are important factors affecting swine farm productivity and animal health and welfare. Respiratory diseases may impair growth rates, contributing to poorer quality meat products, and can increase the need for drug use and the costs associated with swine production [1, 2]. Porcine reproductive and respiratory syndrome virus (PRRSV), influenza A virus (IAV), and *Mycoplasma hyopneumoniae* (*M. hyopneumoniae*) are three of the most significant pathogens affecting swine farm productivity [3]. Despite efforts to eradicate these pathogens and their associated diseases, they continue to be widespread in the world swine population and result in huge economic losses for pork producers. These infectious agents can be detrimental to production on their own, and the presence of multiple infectious agents on-farm can increase the risk for co-infection [4–7] and can also lead to more severe disease than single infection with either agent(s) [8, 9]. These interactions may further exacerbate declines in producer profits and animal welfare [1].

The developing immunity of young pigs can also increase the difficulty in preventing disease on farm. As piglets are weaned from the dam, their levels of maternal antibodies decline, after which pigs begin to produce their own antibodies as they become exposed to pathogens and their immune systems start to develop [10]. This decrease in maternal antibody levels increases the risk for disease in young pigs. While vaccination is a common practice on swine farms, the prevalence of particular pathogens in a herd may change due to many factors including but not limited to proximity to other herds, air quality, biosecurity, genetics, in-feed medication, as well as diet and feeding program [11–14].

Pigs commonly receive a series of starter feeds in order to slowly transition weanlings from expensive, complex diets containing easily digestible ingredients such as milk products and fishmeal to less expensive diets consisting of simpler, plant-based ingredients [15]. This allows pigs to develop the necessary enzymes required for digesting the constituents found in adult diets and reduces post-weaning growth lag [15, 16]. Proper feeding also improves resistance to many bacterial and parasitic infections and shortens recovery times. However, not only is feed the costliest aspect associated with pork production, but nursery diet costs are especially high due to the need for a highly palatable feed that will allow the immature gut of the pig to adapt from an easily digestible milk diet to solid, grain-based feed [16]. Substituting complex proteins in nursery feed for simpler, plant-based proteins may offer cost-saving benefits to producers without sacrificing carcass quality and growth rate [17].

However, humoral and inflammatory responses may be reduced in pigs receiving diets containing higher levels of soy protein which could indicate immune function is compromised due to this type of diet [18]. While previous research has demonstrated that low complexity nursery diets have no significant effect on antibody responses to *Salmonella* [19], further research is still needed in order to better understand the possible effects of nursery diet complexity on antibody responses to other important porcine production-limiting pathogens.

The objectives of this study were: 1) to measure antibody responses to PRRSV, IAV, and *M. hyopneumoniae* in pigs from weaning to the end of the finisher stage; 2) to examine the relationship in antibody responses among those three pathogens; and 3) to determine the association of a nursery diet that uses mostly plant protein (compared to the typical complex animal protein-based diet) with seropositivity to PRRSV, IAV, and *M. hyopneumoniae*.

## Results

### Seropositivity to PRRSV, IAV, and *M. hyopneumoniae* at the pig and group level

Of the 336 pigs tested for all three pathogens at four visits, 24 (7.1%) were seronegative for all three pathogens throughout production, 165 (49.1%) were seropositive for one at least once over the course of production, 124 (36.9%) were seropositive for two, and 23 (6.9%) were seropositive for all three pathogens.

**Fig. 1:**
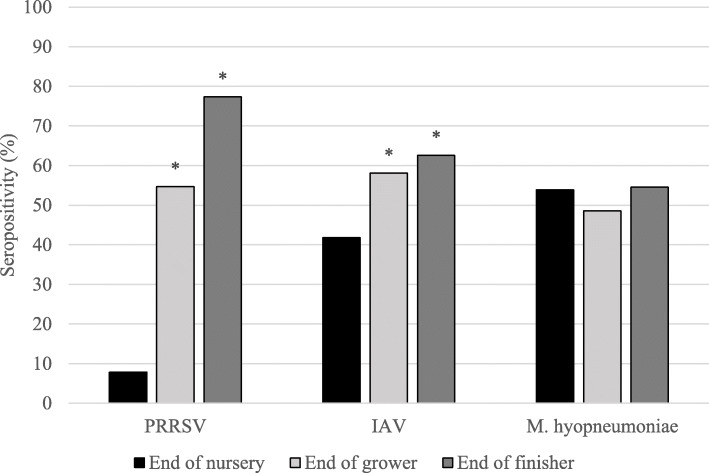
Percentage of pigs seropositive for PRRSV, IAV, and *M. hyopneumoniae* from the end of nursery to the end of finisher. This figure depicts the percentage of pigs that were seropositive for porcine reproductive and respiratory syndrome virus (PRRSV), influenza A virus (IAV), and *Mycoplasma hyopneumoniae* in seropositive groups at each stage of production from the end of nursery to the end of the finisher stage. Note: Two, 9 and 4 groups were classified as seropositive for PRRSV, IAV, and *M. hyopneumoniae,* respectively. *Significantly different from end of nursery (*p* <  0.05)

**Fig. 2:**
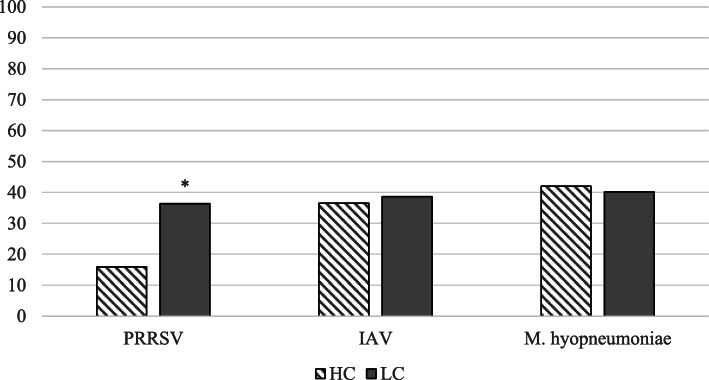
Combination frequency of pigs that were seropositive for PRRSV at different stages of production. This figure displays pigs that were tested at 4 visits and were seropositive for porcine reproductive and respiratory syndrome virus (PRRSV) at least once throughout production. The data are presented as intersections for each combination of production stages in which pigs were seropositive, with seropositivity represented by dots in the horizontal axis. The number of seropositive pigs per intersection are displayed above the corresponding bar and arranged from the largest intersection to the smallest. Dots are connected by a line if pigs were seropositive at more than 1 production stage. For example, the largest number of pigs were seropositive for PRRSV only at weaning, followed by pigs that were only seropositive at the end of the finisher stage, etc.

Figure 1 displays the percentage of pigs that were seropositive for each pathogen from the end of nursery to the end of the finisher stage. Due to the possible confounding effects of passive immunity on the objectives of this study, groups were considered positive for each pathogen if more than 20% of pigs in that group tested seropositive for that pathogen at least once from the end of nursery to the end of the finisher stage. The proportion of seropositivity to each pathogen in pigs fed high or low complexity diet in seropositive groups is shown in Fig. 2. Of the 14 groups, two were classified as seropositive for PRRSV (pig seropositivity: 38.3–65.0%), nine groups were seropositive for IAV (pig seropositivity: 33.3–70.0%), and four were seropositive for *M. hyopneumoniae* (pig seropositivity: 26.7–85.0%) (Table 1). Statistical analysis was then conducted on the positive groups from weaning to the end of the finisher stage at the pig level in order to determine how seropositivity changes over the course of production.

**Table 1:** Pig-level seropositivity to PRRSV, IAV, and *M. hyopneumoniae* in 14 groups of pigs

		% of pigs testing seropositive at least once							
		Farrowing source							
	Cohort	1	2	3	4	5	6	7	8
PRRSV	One	0.0	0.0	0.0	0.0	8.3	38.3	0.0	0.0
	Two	1.7	ND	0.0	0.0	0.0	0.0	ND	65.0
IAV	One	35.2	17.2	33.3	3.3	11.7	13.3	60.0	70.0
	Two	5.0	ND	68.3	51.7	68.3	63.3	ND	70.0
*M. hyo*	One	^a^72.2	8.6	0.0	1.7	85.0	1.7	48.3	11.7
	Two	^a^25.0	ND	1.7	0.0	66.7	1.7	ND	26.7

This table depicts the percentage of individual pigs that were seropositive for porcine reproductive and respiratory syndrome virus (PRRSV), influenza A virus (IAV), or *Mycoplasma hyopneumoniae* at least once in 14 groups of pigs from 8 farrowing sources. A group was considered seropositive if more than 20% of pigs were seropositive at least once from the end of nursery to the end of the finisher stage

*ND* No data (blood samples were not collected)

^a^ Vaccinated for *M. hyopneumoniae*

### Multivariable analysis

**Fig. 3:**
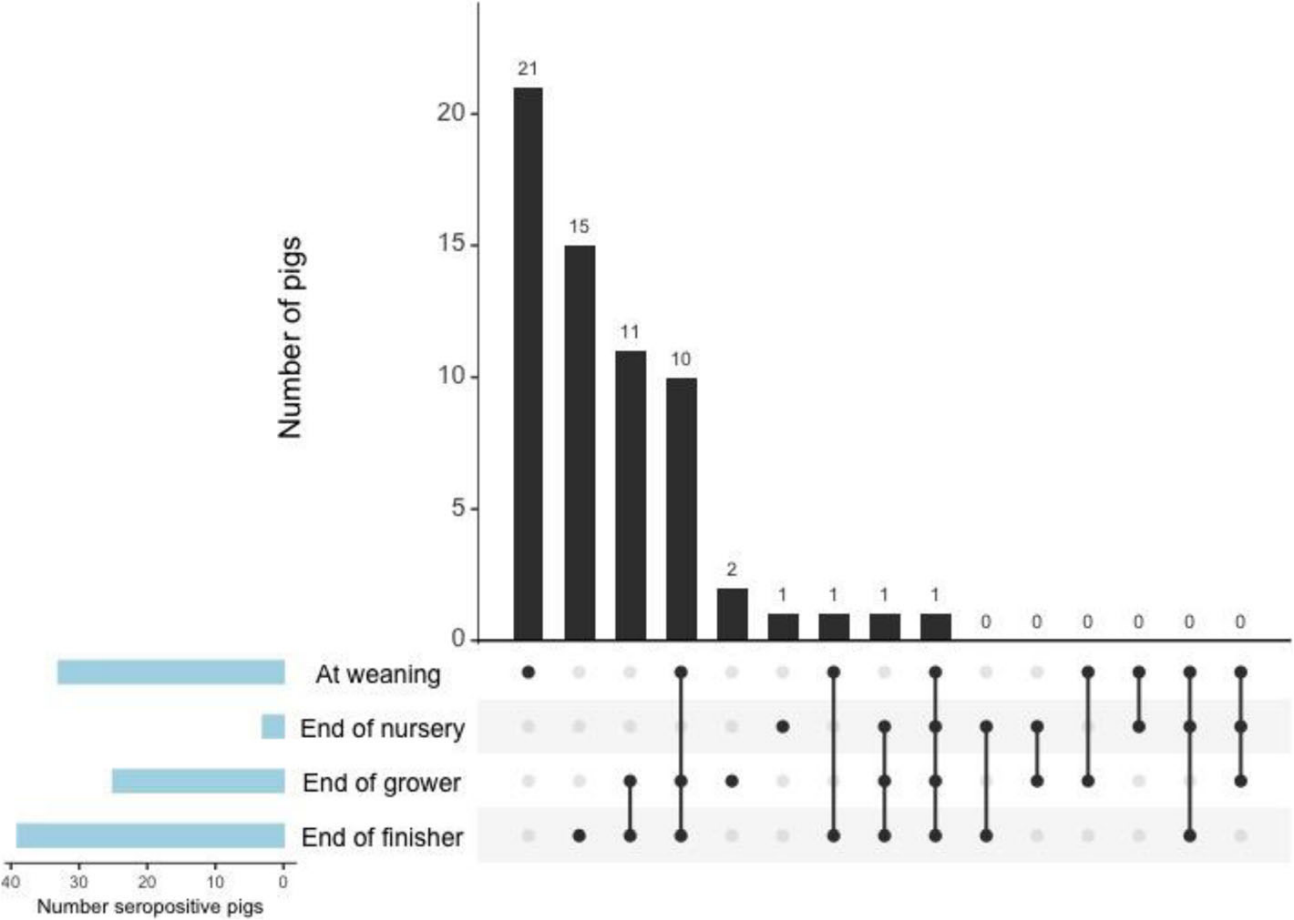
Combination frequency of pigs that were seropositive for IAV at different stages of production. This figure displays pigs that were tested at 4 visits and were seropositive for influenza A virus (IAV) at least once throughout production. The data are presented as intersections for each combination of production stages in which pigs were seropositive, with seropositivity represented by dots in the horizontal axis. The number of seropositive pigs per intersection are displayed above the corresponding bar and arranged from the largest intersection to the smallest. Dots are connected by a line if pigs were seropositive at more than 1 production stage. For example, the largest number of pigs were seropositive for IAV only at weaning, followed by pigs that were seropositive at all four production stages, etc.

**PRRSV.** Pigs from two seropositive groups that had more than 20% of pigs test seropositive for PRRSV at least once over the course of production were included in the multivariable analysis. In the two seropositive groups, seropositivity was likely to increase with age (*p* <  0.001). Pigs fed a low complexity nursery diet were more likely to be seropositive than those fed a high complexity diet (p <  0.001) (Table 2). The combination of production stages in which pigs were seropositive for PRRSV if tested at four visits is shown in Fig. 3. Four pigs from two different farrowing sources were identified as outliers. The best linear unbiased predictors followed a normal distribution and the model met the homoscedasticity assumption.

**Table 2:** Mixed-effects multi-level logistic regression analysis for porcine reproductive and respiratory syndrome virus (PRRSV) seropositivity

Parameter	Odds ratio	Standard error	95% confidence interval	*p*-value
Diet^a^				
HC	Referent			
LC	10.60	3.90	5.18–21.90	< 0 .001
IAV seropositivity				
No	Referent			
Yes	3.03	1.3	1.3–7.0	0.009
Age (weeks)	1.26	0.04	1.18–1.40	< 0 .001

This table displays the mixed-effects multi-level logistic regression analysis for porcine reproductive and respiratory syndrome virus (PRRSV) seropositivity with sow as a random effect in 2 seropositive groups from 2 different farrowing sources that had more than 20% of pigs test seropositive to PRRSV by ELISA

*IAV* influenza A virus

^a^*HC* high complexity diet; *LC* low complexity diet

**Fig. 4:**
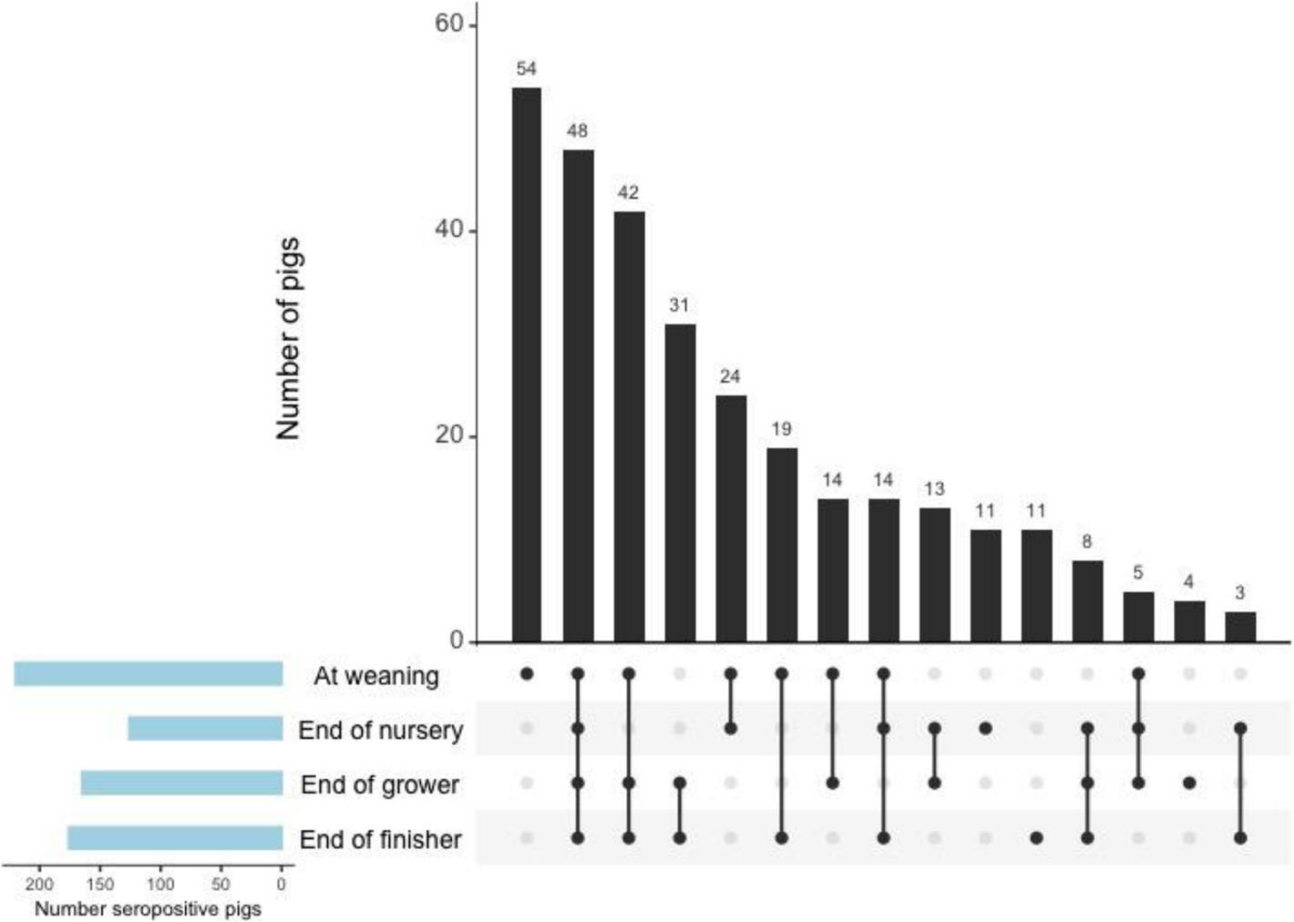
Combination frequency of pigs that were seropositive for *M. hyopneumoniae* at different stages of production. This figure displays pigs that were tested at 4 visits and were seropositive for *Mycoplasma hyopneumoniae* (*M. hyopneumoniae*) at least once throughout production. The data are presented as intersections for each combination of production stages in which pigs were seropositive, with seropositivity represented by dots in the horizontal axis. The number of seropositive pigs per intersection are displayed above the corresponding bar and arranged from the largest intersection to the smallest. Dots are connected by a line if pigs were seropositive at more than 1 production stage. For example, the largest number of pigs were seropositive for *M. hyopneumoniae* only at weaning, followed by pigs that were only seropositive at the end of the finisher stage, etc.

**IAV.** Pigs from nine seropositive groups that had more than 20% of pigs test seropositive for IAV at least once from the end of nursery to the end of finisher were included in the multivariable analysis. Pigs were more likely to be seropositive for IAV at weaning and the end of the grower and finisher stages compared to the end of nursery (*p* <  0.001). Pigs in Cohort Two (born between October and January) were more likely to be seropositive than pigs in Cohort One (born between May and August) (p <  0.001) (Table 3). Pigs that were seropositive for *M. hyopneumoniae* were more likely to be seropositive for IAV (*p* = 0.007). There was no significant association between diet and antibody responses (*p* = 0.17). The variation in pig seropositivity due to farrowing source and sow was 78.3 and 21.7%, respectively. The combination of production stages in which pigs were seropositive for IAV if tested at four visits is shown in Fig. 4. Examining the Pearson residual, nine pigs from 3 different farrowing sources were identified as outliers. The best linear unbiased predictors followed a normal distribution and the model met the homoscedasticity assumption.

**Table 3:** Mixed-effects multi-level logistic regression analysis for influenza A virus (IAV) seropositivity

Parameter	Odds ratio	Standard error	95% confidence interval	p-value
*M. hyo* seropositivity				
No	Referent			
Yes	1.66	0.31	1.15–2.41	0.007
Production stage				
At weaning	3.10	0.54	2.20–4.35	< 0.001
End of nursery	Referent			
End of grower	2.30	0.40	1.63–3.22	< 0.001
End of finisher	2.89	0.52	2.03–4.10	< 0.001
Cohort^a^				
One	Referent			
Two	3.64	0.98	2.15–6.16	< 0.001

This table displays the mixed-effects multi-level logistic regression analysis for influenza A virus (IAV) seropositivity with farrowing source and sow as random effects in 9 seropositive groups that had more than 20% of pigs test seropositive to IAV by ELISA

*M. hyo* = *Mycoplasma hyopneumoniae*

^a^Cohort One: Pigs were born between May and August; Cohort Two: Pigs were born between October and January

#### *M. hyopneumoniae*

**Fig. 5:**
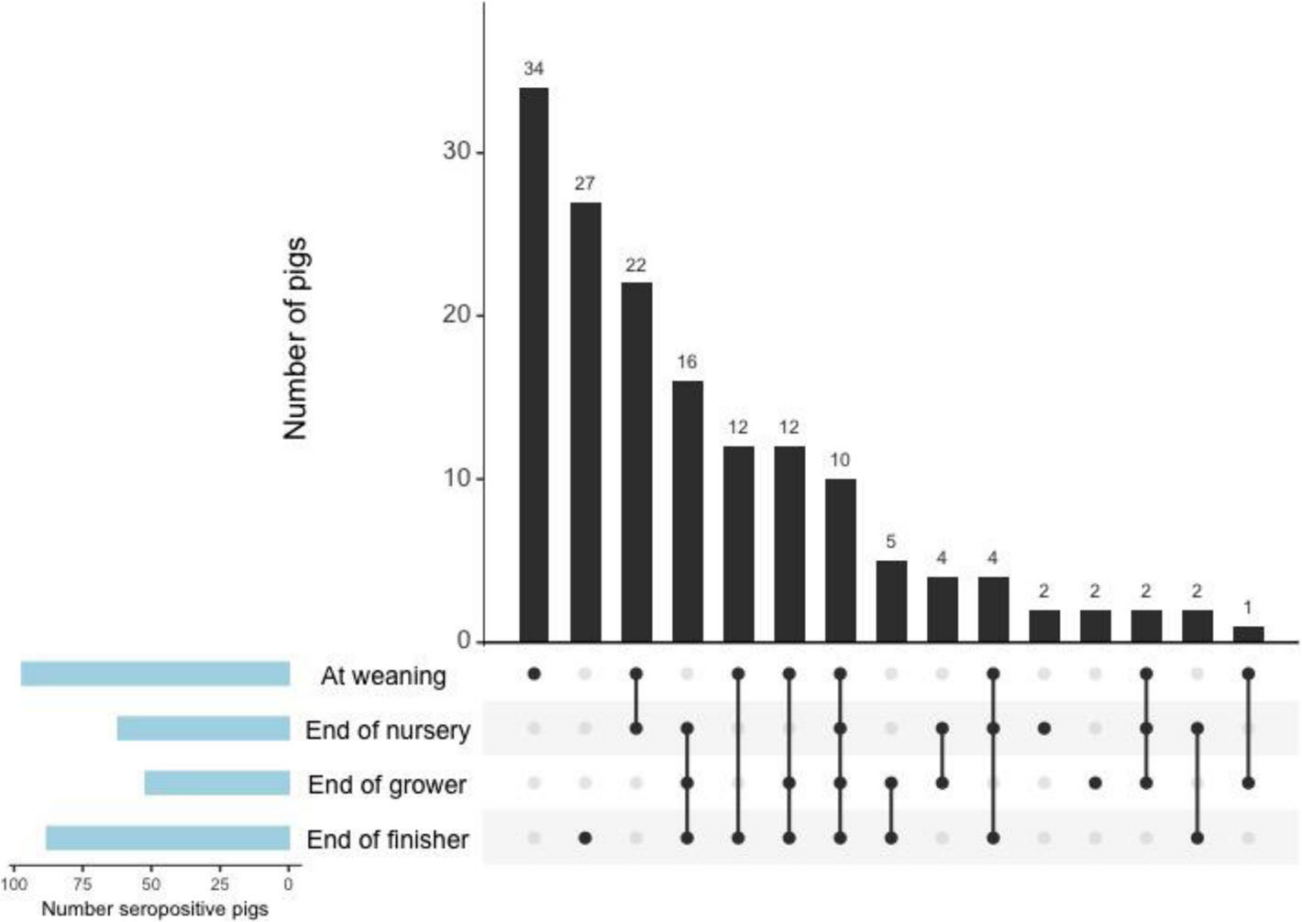
PRRSV, IAV, and *M. hyopneumoniae* seropositivity in pigs fed high or low complexity nursery diet. This figure depicts the proportion of seropositivity to porcine reproductive and respiratory syndrome virus (PRRSV), influenza A virus (IAV), and *Mycoplasma hyopneumoniae* among pigs fed high or low complexity diet in seropositive groups. Note: 2, 9, and 4 groups were classified as seropositive for PRRSV, IAV, and *M. hyopneumoniae*, respectively. *p <  0.05

Six groups had more than 20% of pigs test seropositive at least once for *M. hyopneumoniae* from the end of nursery to the end of the finisher stage. However, two seropositive groups from one farrowing source were excluded from the multivariable analysis as sows and pigs in those groups were vaccinated for *M. hyopneumoniae*. Therefore, only pigs from four seropositive groups from three different farrowing sources were included in the multivariable analysis. Within these groups, pigs that were seropositive for IAV (*p* <  0.001) were more likely to be seropositive for *M. hyopneumoniae* (Table 4). Older pigs were less likely to test seropositive compared to younger pigs (p <  0.001). The farrowing source was associated with seropositivity to *M. hyopneumoniae* (p <  0.001). There was no significant association between diet and antibody responses to *M. hyopneumoniae* (*p* = 0.97). The combination of production stages in which pigs were seropositive for *M. hyopneumoniae* if tested at four visits is shown in Fig. 5. Plotting the Pearson residuals against the predictive probability of *M. hyopneumoniae* seropositivity, six pigs from two different farrowing sources were identified as outliers. The best linear unbiased predictors followed a normal distribution and the model met the homoscedasticity assumption.

**Table 4:** Mixed-effects multi-level logistic regression analysis for *Mycoplasma hyopneumoniae*

Parameter	Odds ratio	Standard error	95% confidence interval	p-value
IAV seropositivity				
No	Referent			
Yes	2.5	0.62	1.5–4.1	< 0.001
Farrowing source				
5	Referent			
7	0.34	0.16	0.14–0.86	0.022
8	0.08	0.04	0.03–0.22	< 0.001
Age (weeks)	0.93	0.015	0.90–0.96	< 0.001

This table displays the mixed-effects multi-level logistic regression analysis for *Mycoplasma hyopneumoniae* seropositivity with sow as a random effect in 4 seropositive groups from 3 farrowing sources. Six groups had more than 20% of pigs test seropositive for *M. hyopneumoniae* at least once from the end of nursery to the end of finisher by ELISA. However, 2 seropositive groups from 1 farrowing source were excluded from the multivariable analysis as sows and pigs were vaccinated for *M. hyopneumoniae*

*IAV* Influenza A virus

^a^Cohort One: Pigs were born between May and August; Cohort Two: Pigs were born between October and January

## Discussion

One objective of this study was to investigate antibody responses to porcine reproductive and respiratory syndrome virus, influenza A virus, and *Mycoplasma hyopneumoniae* in pigs at different stages of production. In general, seropositivity indicates that an animal has either absorbed maternally derived antibodies or has been exposed to infectious agents through natural infection or vaccination and was able to mount a robust immune response. Seropositivity proportions were high at weaning for all three pathogens, likely due to the absorption of antibodies through the sow’s colostrum and milk [20, 21], and low again at the end of the nursery stage. Seropositivity was more likely to increase with age for PRRSV and was also more likely to be higher at the end of the grower and finisher stages compared to the end of the nursery stage for IAV. The decline in seropositivity observed from weaning to the end of nursery in this study indicates the loss of maternal antibodies [10], which also suggests that pigs are particularly susceptible to infection post-weaning. Except for one farrowing source and its two out-going groups, which were vaccinated for *M. hyopneumoniae*, there was no vaccination to any of the three pathogens in the farrowing sources or their out-going groups. Thus, it is largely assumed that increases in antibody responses post-weaning were the result of natural infection.

Older pigs were less likely to be seropositive for *M. hyopneumoniae* than younger pigs, and this decreased likelihood for *M. hyopneumoniae* seropositivity may indicate the vulnerability of weanlings to infection, as antibody responses did not seem to increase significantly with age. This may also indicate the lack of *M. hyopneumoniae* infections in the later production stages. Due to the tendency of *M. hyopneumoniae* to produce chronic infections in the host, the assumption is that after the decline of maternal antibodies, the young pigs mount a slower immune response [22] or that *M. hyopneumoniae* itself is slower to spread and thus antibody responses appear delayed [23].

The second objective of this study was to determine if infection with one pathogen increases the likelihood for seropositivity to another. It was found that pigs in seropositive groups that were seropositive for IAV were more likely to be seropositive for PRRSV and *M. hyopneumoniae*. Additionally, pigs in seropositive groups that were seropositive for *M. hyopneumoniae* were more likely to be seropositive for IAV. The present study did not determine if co-infection could produce more severe disease, but these results have been reported in the past [7, 24]. Additionally, while managerial factors, such as pig density and pig flow, would affect the spread of disease on farm, it is possible that infection with one agent would increase susceptibility to other agent(s). This suggests that while controlling for the presence of one pathogen is important, in order to prevent more severe disease, control strategies should be directed towards preventing co-infection. Understanding which pathogens are a threat on a farm-specific basis using techniques such as ELISA may help in reducing the detrimental effects of co-infection.

The third objective of this study was to investigate whether a low complexity nursery diet with a higher amount of fibre provided by corn and soybean was associated with antibody responses to PRRSV, IAV, and *M. hyopneumoniae*. Pigs fed a low complexity nursery diet were more likely to be seropositive for PRRSV; however, there was no significant association between nursery diet and IAV or *M. hyopneumoniae* seropositivity. This experimental low complexity diet has also been previously found to have no effect on antibody responses to *Salmonella* [19]. These results may suggest that the low complexity diet increased the susceptibility of pigs to PRRS virus but had no effect on susceptibility to IAV, *M. hyopneumoniae*, or *Salmonella*. Alternatively, these results may indicate that the LC diet elevated the immune response to PRRSV while having no effect on the immune response to IAV, *M. hyopneumoniae,* and *Salmonella*. From a production standpoint, the lack of association between antibody responses to IAV and *M. hyopneumoniae* and feeding the low complexity nursery diet is promising, as it may encourage the implementation of these cost-saving diets with no adverse effects on herd health. However, the results in this study should be interpreted with caution and need to be examined more thoroughly using control challenge studies. Further investigation into the effects of nursery diet complexity on antibody responses to other notable porcine pathogens may help shed more light on the effects of diet on immune development. Other branches of the immune system, such as cell-mediated immune responses, should also be examined. Additionally, the possible effect of diet complexity on the digestive health on the animals requires further scrutiny to ensure adverse effects on digestion do not occur as a result of feeding lower complexity nursery diets.

While seropositivity at the pig level was relatively high, a proportion of pigs remained seronegative throughout all stages of production. This indicates either that these pigs were never exposed to the infectious agents; that animals were exposed but the pathogens were unable to bypass the innate immune system in order to establish infection and activate the adaptive immune system; that an immune response was generated but was not robust enough to be read as seropositive by the ELISA kits; or that pigs had not yet seroconverted at the time of sample collection. However, there may have also been some variation in results based on the ELISA kits used for antibody detection. The IDEXX ELISA kits have been found to have 100% sensitivity and 99.9% specificity for PRRSV [25]; 86 and 89% for IAV [26]; and relatively low sensitivity (63%) but high specificity (100%) for *M. hyopneumoniae* [22]. However, as noted by Erlandson et al. [18], the low sensitivity of the *M. hyopneumoniae* test is likely due to the nature of the infectious agent and the slow immune response produced by *M. hyopneumoniae* rather than the ability of the ELISA kits to identify seropositive pigs. The low sensitivity could also be due to the slow transmission of *M. hyopneumoniae*, which would also delay the seroconversion [23]. This seems to suggest that the pathogen is still present throughout production but the propagation between animals is slow or the immune response itself is delayed. This low sensitivity may have increased the chance of false negatives in this study but considering a pig “seropositive” if it tested seropositive at least once over the course of production likely worked to counteract this issue. Further, the ELISA kits used in this study could only detect IgG antibodies, which may have resulted in more recent infections at one specific sampling occasion being left undetected (false negative). However, these infections could eventually be detected over the next sampling time points.

The ELISA kits used in this study were unable to differentiate between antibody responses to natural infections and vaccination. However, because only one farrowing source was vaccinated for *M. hyopneumoniae* and the corresponding groups were not included in the analyses, the seropositivity observed in the high seropositivity groups can be largely assumed to be from maternal antibodies in the early stages of production and natural infection later in life. These results may help to encourage vaccination in post-weaning pigs, when the interaction between maternal antibodies and vaccine antigens is minimized [10].

## Conclusion

Understanding periods of vulnerability on farm is important in order to develop site-specific methods of disease prevention and control. Monitoring frequently for changes in the seroprevalence of pathogens on swine farms may help to confer broader protection and improve animal health and welfare. Finally, while further research is needed to investigate the association between low complexity nursery diets and other important health factors, such as other branches of the immune system and the effects on digestive health, this study suggests that low complexity nursery diets, which offer cost-saving incentives, may be beneficial on farms with low disease pressures.

## Methods

### Farrowing source and pig selection

The farrowing source and pig selection have been described previously [19, 27]. Briefly, fourteen groups of 54–60 pigs from a convenience sample originating from eight farrowing sources in Southwestern Ontario were selected. Two cohorts (Cohort One and Two) were included in the study from six of the eight farrowing sources, while the other two included only one cohort (Cohort One). Pigs in Cohort One were born between May and August, while pigs in Cohort Two were born between October and January. All sources but one utilized off-site nursery and finishing, while the other was farrow-to-finish for Cohort One and off-site finisher for Cohort Two.

### Diet

Pigs in each group were divided equally to receive either a standard animal-protein based (high complexity, HC) diet (n1 = 27–30) or an experimental plant (soybean and corn)- protein based, (low complexity, LC) diet (n2 = 27–30) during the nursery phase. Both diets were formulated with similar nutrient levels but different ingredient composition. The high-complexity diet contained whey, fishmeal, and soy protein concentrate, while in the low-complexity diet the majority of the protein was primarily sourced from corn and soybean meal [17, 28]. A detailed breakdown of ingredient composition and feeding schedule has been published previously [14]. The diets were administered in three phases and were available ad libitum, with phases I, II, and III being fed for an average of 9, 15, and 14 days, respectively. At all other stages, all pigs were fed common grower and finisher diets according to the practices of the individual farms. Pigs were tagged to ensure both that they received their intended diet and that samples could be taken at multiple visits.

### Questionnaire

A questionnaire was used to obtain information regarding farm management practices (Additional File 1).

### Sample collection

Blood samples were collected from all pigs at weaning and at the end of the nursery, grower, and finisher stages in all 14 groups except for one, where samples were not collected at the end of the finisher stage. Samples were collected between May 2014 and June 2016. Average ages of pigs at each sampling point were 26, 61, 106, and 145 days at weaning, the end of nursery, end of grower, and end of finisher, respectively. Blood samples were collected from either the jugular vein or suborbital sinus and centrifuged at 1500 x g for 20 min.

### Enzyme-linked immunosorbent assay (ELISA)

Sera were analyzed for the presence of PRRSV, IAV, and *M. hyopneumoniae* antibodies using three commercially available ELISA Kits (Porcine Reproductive and Respiratory Syndrome Virus Antibody Test Kit; Swine Influenza Virus Antibody Test Kit; Mycoplasma Hyopneumoniae Antibody Test Kit; IDEXX Laboratories, Inc., Westbrook, Maine, USA) as per the manufacturer’s instructions. The sensitivities and specificities for each ELISA were: 100 and 99.9% for PRRSV; 86 and 89% for IAV; and 63 and 100% for *M. hyopneumoniae*. A pig was considered seropositive for a specific pathogen if it was seropositive at least once from the end of nursery to the end of the finisher stage for that pathogen. However, in order to increase the test specificity at the group level, a group was classified as positive for a pathogen if at least 20% of the pigs in the group were seropositive for that pathogen at least once from the end of nursery to the end of the finisher stage.

A sample-to-positive (*S/P*) ratio for PRRSV antibodies was calculated as follows:

$$ \mathrm{S}/\mathrm{P}=\frac{\mathrm{Sample}\ \mathrm{absorbance}\ (650)-{\mathrm{Mean}}_{\mathrm{negative}\ \mathrm{control}}}{{\mathrm{Mean}}_{\mathrm{positive}\ \mathrm{control}}-{\mathrm{Mean}}_{\mathrm{negative}\ \mathrm{control}}} $$

A pig was considered seropositive for PRRSV if the *S/P* ratio was ≥0.4.

A sample-to-negative (*S/N*) ratio for IAV was calculated as follows:

control$$ \mathrm{control} $$ $$ \mathrm{S}/\mathrm{N}=\frac{\mathrm{Sample}\ \mathrm{absorbance}\ (650)}{{\mathrm{Mean}}_{\mathrm{negative}\ \mathrm{control}}} $$

A pig was considered seropositive for IAV if the *S/N* ratio was < 0.6.

A *S/P* ratio for *M. hyopneumoniae* was calculated as follows:

$$ S/P=\frac{\mathrm{Sample}\ \mathrm{absorbance}\ (650)-{\mathrm{Mean}}_{\mathrm{negative}\ \mathrm{control}}}{{\mathrm{Mean}}_{\mathrm{positive}\ \mathrm{control}}-{\mathrm{Mean}}_{\mathrm{negative}\ \mathrm{control}}} $$

A pig was considered seropositive for *M. hyopneumoniae* if the S/P ratio was > 0.4.

### Data analysis

Data were cleaned in Excel (Microsoft 2016, Redmond, Washington, USA) and transferred to Stata (Stata/MP-13 StataCorp, College Station, Texas, USA) for analysis. Samples from weaning to the end of the finisher stage were included in the data analysis from only those pigs within seropositive groups. A mixed-effects multi-level logistic regression method with farrowing source and sow as random effects to account for clustering was used to compare IAV and *M. hyopneumoniae* seropositivity in pigs at different stages of production. For PRRSV, only sow was included as a random effect because only two groups were seropositive and included in the data analysis. Two *M. hyopneumoniae* seropositive groups from farrowing source #1 were excluded from data analysis as sows and out-going pigs were vaccinated for *M. hyopneumoniae*. The independent variables considered as fixed effects were nursery diet (HC/LC), cohort (i.e. Cohort One or summer: pigs were born between May and August; Cohort Two or winter: pigs were born between October and January), age, production stage (end of nursery/end of grower/end of finisher), and seropositivity to other pathogens of interest for the present study (yes/no). While groups were classified as seropositive if at least 20% of pigs in that group were seropositive at least once from the end of nursery to the end of the finisher stage, statistical analyses were performed at the pig level in those seropositive groups, including samples at weaning to determine changes in seropositivity over the course of production. Univariable analysis for the association between the independent variables and pig seropositivity to each pathogen was first evaluated by a single logistic regression method, and variables with a *p* <  0.2 were considered for inclusion in the multivariable analysis. Models were then built using a manual forward stepwise approach and variables were included in the final models if *p* <  0.05. The normal quantiles plots were created to evaluate the normality assumption for each model. Also, in order to examine homoscedasticity of the best linear unbiased predictors (BLUPS) the BLUPS were plotted against the predicted log odds of the outcome in each model. The Pearson residuals were generated and used to identify outliers.

## Supplementary Information

**Additional file 1. **Questionnaire**.** This questionnaire was developed and used to collect information about management practices in place on participating farms.

## Data Availability

The data used and analyzed during the current study are available from the corresponding author upon request.

## Abbreviations

ELISA: Enzyme-linked immunosorbent assay
HC: High complexity
IAV: Influenza A virus
LC: Low complexity
PRRSV: Porcine reproductive and respiratory syndrome virus
S/N: Sample-to-negative
S/P: Sample-to-positive
