# Supplementary material for: Antibody responses to porcine reproductive and respiratory syndrome virus, influenza A virus, and Mycoplasma hyopneumoniae from weaning to the end of the finisher stage in fourteen groups of pigs in Ontario, Canada
Source: BMC Vet Res. 2021 Feb 17;17:82. doi: 10.1186/s12917-021-02756-6 (PMC7887834; doi:10.1186/s12917-021-02756-6)
Supplement: Supplementary file 1 — Additional file 1. Questionnaire. This questionnaire was developed and used to collect information about management practices in place on participating farms. [file 12917_2021_2756_MOESM1_ESM.docx]

**Farm Management Survey Farm ID:**

**Part 1 – Farm**

1. Type of farm operation: ________________________________________________________
2. Number of animals on farm

SOWS: _____________ SUCKLING: ___________

NURSERY: ___________ GROWERS: ___________

FINISHERS: ___________ Boers: __________

Replacement gilts: _________

1. Number of rooms on farm

FARROWING: ___________ NURSERY: __________

GROWER: _____________ FINISHER: __________

DRY SOW: ____________ GILT: ___________

BOAR: ___________ ISOLATION/OTHER: _____________________

1. Type of Operation:
   1. Closed operation
   2. Open operation
2. Pig Flow
   1. Continuous flow
   2. All in all out
      1. Entire barn
      2. Nursery room/pens
      3. Grower finisher room/pens
      4. For certain stages only (please list): ________________________________

_____________________________________________________________

1. Source of gilt replacement: ____________________________________________________

**Part 2 – Biosecurity**

1. Shower in and out? Yes No Other: ____________
2. Wash/clean between batches? Yes No Other: ____________
   1. If yes, which rooms/stages?
      1. Farrowing iv. Grower
      2. Gestation v. Finisher
      3. Weaners vi. Other: _________________________
3. Down time between batches? Yes No Other: ____________
   1. If yes, how much downtime? _______________________________________________

_______________________________________________________________________

- 1. Same downtime between each stage?

1. Please list any other biosecurity features of your operation that may have been missed by this survey:

**Part 3 – Feed**

1. Feed supplier? ______________________________________________________
2. Type of feed?
   1. Pellet feed
   2. Mash
   3. Liquid feeding
   4. Wet/dry feed
   5. Other: ___________________________________________
3. Do you use creep feed?
   1. Yes
   2. No
4. Do you use hormones or growth promoters in your operation?
   1. Yes (please specify):_______________________________________________________
   2. No
5. In-feed medications?
   1. Yes (please specify): _______________________________________________________
   2. No
6. In-water medications?
   1. Yes (please specify): _______________________________________________________
   2. No
7. Does your feed contain dried blood plasma as a source of protein?
   1. Yes
   2. No, never
   3. No, but used in the past (why did you stop? ___________________________________)

**Part 4 – Health**

1. Please describe your vaccination programs.
2. PRRSV
   1. Positive Last test date: _________________
   2. Negative
3. Have you had any outbreaks in the past year? If yes, please specify.

___________________________________________________________________________

___________________________________________________________________________

🡪 Were these outbreaks suspected or confirmed? _______________________________

1. Has your farm previously been tested for salmonella?
   1. No
   2. Yes (Last test date: _________________ Result: _____________________ )

**Part 5 (To be completed by surveyor)**

**Breed information:**

**Barn schematic :**
